# 11β,13-Dihydro­lactucin-8-*O*-acetate hemihydrate

**DOI:** 10.1107/S160053680903829X

**Published:** 2009-09-30

**Authors:** Chris F. Fronczek, Marco L. Gomez-Barrios, Nikolaus H. Fischer, Frank R. Fronczek

**Affiliations:** a Department of Chemistry, Louisiana State University, Baton Rouge, LA 70803-1804, USA

## Abstract

The title structure (systematic name: 9-hydroxy­methyl-3,6-di­methyl-3-methyl­ene-2,7-dioxo-3,3a,4,5,9a,9b-hexa­hydro­azu­leno[4,5-*b*]furan-4-yl acetate hemihydrate), C_17_H_20_O_6_·0.5H_2_O, from *Lactuca floridana*, has two independent sesquiterpene lactone mol­ecules in the asymmetric unit. Both have their seven-membered rings in the chair conformation. In the crystal, the OH groups and the water mol­ecule form classical O—H⋯O hydrogen bonds with O⋯O distances in the range 2.6750 (17)–2.8160 (18) Å.

## Related literature

For phytochemical reports of the title compound, see: Bohlmann *et al.* (1981 ▶); Djordjevic *et al.* (2004 ▶); Sarg *et al.* (1982 ▶); Song *et al.* (1995 ▶). The crystal structures of several related compounds have been reported: 8-α-hydroxy­achillin (Campos *et al.*, 1989 ▶); matricarin (Parvez *et al.*, 2002 ▶); lactucin (Ruban *et al.*, 1978 ▶); lactucopicrin (Ren *et al.*, 2003 ▶); absolute configuration of sesquiterpene lactones Fischer *et al.* (1979 ▶). For analysis of Bijvoet pairs, see: Hooft *et al.* (2008 ▶). 
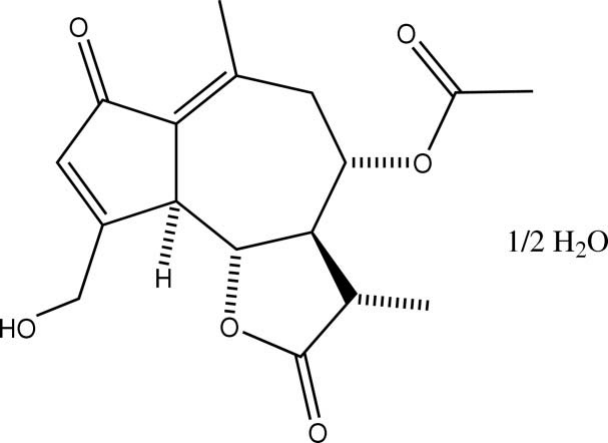


## Experimental

### 

#### Crystal data

C_17_H_20_O_6_·0.5H_2_O
*M* *_r_* = 329.34
Monoclinic, 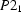

*a* = 10.9276 (5) Å
*b* = 7.4658 (5) Å
*c* = 19.8571 (10) Å
β = 100.850 (5)°
*V* = 1591.05 (15) Å^3^
*Z* = 4
Cu *K*α radiation
μ = 0.88 mm^−1^
*T* = 90 K
0.27 × 0.17 × 0.10 mm

#### Data collection

Bruker Kappa APEXII CCD area-detector diffractometer
Absorption correction: multi-scan (*SADABS*; Sheldrick, 2004 ▶) *T*_min_ = 0.796, *T*_max_ = 0.917
23565 measured reflections
5642 independent reflections
5513 reflections with *I* > 2σ(*I*)
*R*_int_ = 0.031

#### Refinement

*R*[*F* ^2^ > 2σ(*F* ^2^)] = 0.027
*wR*(*F* ^2^) = 0.068
*S* = 1.03
5642 reflections
445 parameters
1 restraint
H atoms treated by a mixture of independent and constrained refinement
Δρ_max_ = 0.20 e Å^−3^
Δρ_min_ = −0.14 e Å^−3^
Absolute structure: Flack (1983 ▶), 2516 Friedel pairs
Flack parameter: −0.01 (10)

### 

Data collection: *APEX2* (Bruker, 2006 ▶); cell refinement: *SAINT* (Bruker, 2006 ▶); data reduction: *SAINT*; program(s) used to solve structure: *SHELXS97* (Sheldrick, 2008 ▶); program(s) used to refine structure: *SHELXL97* (Sheldrick, 2008 ▶); molecular graphics: *ORTEP-3 for Windows* (Farrugia, 1997 ▶); software used to prepare material for publication: *SHELXTL*.

## Supplementary Material

Crystal structure: contains datablocks global, I. DOI: 10.1107/S160053680903829X/pv2210sup1.cif

Structure factors: contains datablocks I. DOI: 10.1107/S160053680903829X/pv2210Isup2.hkl

Additional supplementary materials:  crystallographic information; 3D view; checkCIF report

## Figures and Tables

**Table 1:** Hydrogen-bond geometry (Å, °)

*D*—H⋯*A*	*D*—H	H⋯*A*	*D*⋯*A*	*D*—H⋯*A*
O6—H6*O*⋯O7	0.86 (2)	1.85 (2)	2.6750 (17)	160 (2)
O6*A*—H60*A*⋯O6	0.85 (2)	1.91 (2)	2.7593 (17)	174 (2)
O7—H72⋯O5*A*^i^	0.89 (2)	1.95 (2)	2.8160 (18)	165 (2)
O7—H71⋯O6*A*^ii^	0.89 (3)	1.86 (3)	2.7397 (18)	176 (3)

Symmetry codes: (i) 
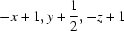
; (ii) 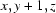.

**Fig. 1.**
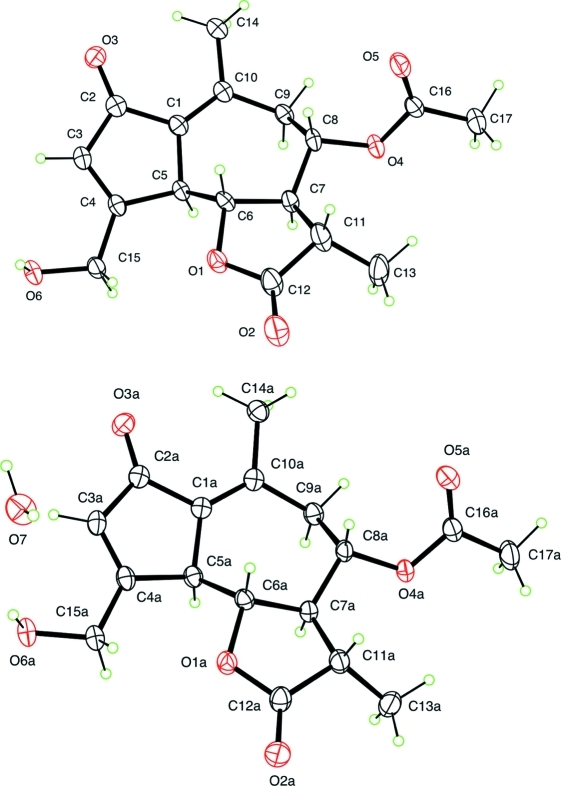
Ellipsoids at the 50% probability level, with H atoms having arbitrary radius. Both molecules are shown in the same orientation.

## supplementary crystallographic information

### Comment

Hairy root cultures of blue-flowered lettice, *Lactuca floridana*, tribe Lactuceae (Asteraceae) are useful for the study of the biosynthesis of guaianolide-type sesquiterpene lactones (Song *et al.*, 1995). The title guaianolide was isolated from *L. floridana* and crystallized as the hemihydrate.

The structures of both independent molecules are shown in Fig. 1. The conformations of (1) and (1 A) are very similar. Both seven-membered rings form chair conformations where atoms C5/C6/C8/C9 are nearly coplanar (maximum deviation 0.022 (1) Å for molecule 1 and 0.024 (1) Å for 1 A). Atom C1 is 0.991 (1) Å above the plane, C7 is 0.751 (1) Å below, and C10 is 1.029 (1) Å above. Atom C1A is 0.881 (1) Å above the plane, C7A is 0.773 (1) Å below, and C10A is 0.965 (1) Å above. Lactone ring (C6/C7/C11/C12/O1) has the C7 envelope conformation, with C7 0.582 (1) Å out of the best plane of the other four (maximum deviation 0.022 (1) Å. Lactone ring (C6A/C7A/C11A/C12A/O1A) also has the C7 envelope conformation, with C7A showing deviation 0.580 (1) Å, and maximum deviation 0.007 (1) Å for the other four. The other 5-membered rings (C1—C5 and C1a—C5A,) are essentially planar (maximum deviations 0.011 (1) Å and 0.041 (1) Å, respectively). This conformation is similar to that seen in matricarin (Parvez *et al.*, 2002), which differs only by lacking the OH group at C15.

Hydrogen bonding involves the OH groups, the water molecule, and the acetate CO group, and forms double-strand chains along [0 1 0]. In each chain, the alternation of hydrogen bonds is (O6A—H···O6—H···H_2_O···)_n_.

The absolute configuration was determined by refinement of the Flack (1983) parameter, based on resonant scattering of the light atoms. It agrees with that of lactucin (Ruban *et al.*, 1978) and with the accepted configuration of sesquiterpene lactones from higher plants (Fischer *et al.*, 1979). Analysis of the Bijvoet pairs using the method of Hooft *et al.* (2008) yielded *y* = 0.03 (4) for this structure, confirming the absolute configuration.

### Experimental

Isolation of the title compound from *Lactuca floridana* has been described (Bohlmann *et al.*, 1981; Song *et al.*, 1995). Crystals were grown by evaporation from ethyl acetate.

### Refinement

H atoms on C were placed in idealized positions with C—H distances 0.95 - 1.00 Å and thereafter treated as riding. Coordinates for the H atoms on O were refined. *U*_iso_ for H was assigned as 1.2 times *U*_eq_ of the attached atoms (1.5 for methyl and OH). A torsional parameter was refined for each methyl group.

### Crystal data

**Table:**

C_17_H_20_O_6_·0.5H_2_O	*F*(000) = 700
*M**_r_* = 329.34	*D*_x_ = 1.375 Mg m^−^^3^
Monoclinic, *P*2_1_	Cu *K*α radiation, λ = 1.54178 Å
Hall symbol: P 2yb	Cell parameters from 9656 reflections
*a* = 10.9276 (5) Å	θ = 2.3–68.3°
*b* = 7.4658 (5) Å	µ = 0.88 mm^−^^1^
*c* = 19.8571 (10) Å	*T* = 90 K
β = 100.850 (5)°	Plate, colorless
*V* = 1591.05 (15) Å^3^	0.27 × 0.17 × 0.10 mm
*Z* = 4	

### Data collection

**Table:**

Bruker Kappa APEXII CCD area-detector diffractometer	5642 independent reflections
Radiation source: fine-focus sealed tube	5513 reflections with *I* > 2σ(*I*)
graphite	*R*_int_ = 0.031
φ and ω scans	θ_max_ = 69.0°, θ_min_ = 2.3°
Absorption correction: multi-scan (*SADABS*; Sheldrick, 2004)	*h* = −12→13
*T*_min_ = 0.796, *T*_max_ = 0.917	*k* = −9→8
23565 measured reflections	*l* = −23→23

### Refinement

**Table:**

Refinement on *F*^2^	Hydrogen site location: inferred from neighbouring sites
Least-squares matrix: full	H atoms treated by a mixture of independent and constrained refinement
*R*[*F*^2^ > 2σ(*F*^2^)] = 0.027	*w* = 1/[σ^2^(*F*_o_^2^) + (0.0314*P*)^2^ + 0.4082*P*] where *P* = (*F*_o_^2^ + 2*F*_c_^2^)/3
*wR*(*F*^2^) = 0.068	(Δ/σ)_max_ = 0.001
*S* = 1.03	Δρ_max_ = 0.20 e Å^−^^3^
5642 reflections	Δρ_min_ = −0.14 e Å^−^^3^
445 parameters	Extinction correction: *SHELXL97* (Sheldrick, 2008), Fc^*^=kFc[1+0.001xFc^2^λ^3^/sin(2θ)]^-1/4^
1 restraint	Extinction coefficient: 0.00101 (13)
Primary atom site location: structure-invariant direct methods	Absolute structure: Flack (1983), 2516 Friedel pairs
Secondary atom site location: difference Fourier map	Flack parameter: −0.01 (10)

### Special details

**Table:**

Geometry. All e.s.d.'s (except the e.s.d. in the dihedral angle between two l.s. planes) are estimated using the full covariance matrix. The cell e.s.d.'s are taken into account individually in the estimation of e.s.d.'s in distances, angles and torsion angles; correlations between e.s.d.'s in cell parameters are only used when they are defined by crystal symmetry. An approximate (isotropic) treatment of cell e.s.d.'s is used for estimating e.s.d.'s involving l.s. planes.
Refinement. Refinement of *F*^2^ against ALL reflections. The weighted *R*-factor *wR* and goodness of fit *S* are based on *F*^2^, conventional *R*-factors *R* are based on *F*, with *F* set to zero for negative *F*^2^. The threshold expression of *F*^2^ > σ(*F*^2^) is used only for calculating *R*-factors(gt) *etc*. and is not relevant to the choice of reflections for refinement. *R*-factors based on *F*^2^ are statistically about twice as large as those based on *F*, and *R*- factors based on ALL data will be even larger.

### Fractional atomic coordinates and isotropic or equivalent isotropic displacement parameters (Å^2^)

**Table:**

	*x*	*y*	*z*	*U*_iso_*/*U*_eq_	
O1	0.36074 (9)	0.72760 (16)	0.04034 (5)	0.0256 (2)	
O2	0.54683 (12)	0.8177 (2)	0.02323 (6)	0.0473 (4)	
O3	−0.15403 (9)	0.63628 (15)	0.05240 (5)	0.0255 (2)	
O4	0.19069 (10)	0.67077 (15)	−0.19419 (5)	0.0240 (2)	
O5	0.07993 (12)	0.91019 (18)	−0.23883 (6)	0.0345 (3)	
O6	0.25725 (10)	0.54254 (16)	0.22516 (5)	0.0252 (2)	
H6O	0.2544 (19)	0.651 (3)	0.2400 (10)	0.038*	
C1	0.02479 (13)	0.5810 (2)	−0.00331 (8)	0.0203 (3)	
C2	−0.04236 (14)	0.61236 (19)	0.05490 (7)	0.0211 (3)	
C3	0.05145 (14)	0.6056 (2)	0.11802 (7)	0.0216 (3)	
H3	0.0338	0.6189	0.1628	0.026*	
C4	0.16502 (14)	0.5782 (2)	0.10450 (7)	0.0196 (3)	
C5	0.16294 (13)	0.5614 (2)	0.02770 (7)	0.0191 (3)	
H5	0.1933	0.4402	0.0170	0.023*	
C6	0.23650 (13)	0.7063 (2)	−0.00135 (6)	0.0201 (3)	
H6	0.1907	0.8225	−0.0025	0.024*	
C7	0.26251 (13)	0.6665 (2)	−0.07304 (7)	0.0210 (3)	
H7	0.2901	0.5390	−0.0742	0.025*	
C8	0.14873 (13)	0.6930 (2)	−0.12911 (6)	0.0210 (3)	
H8	0.1133	0.8156	−0.1260	0.025*	
C9	0.04910 (14)	0.5504 (2)	−0.12638 (7)	0.0221 (3)	
H9A	−0.0085	0.5477	−0.1713	0.026*	
H9B	0.0903	0.4319	−0.1195	0.026*	
C10	−0.02665 (14)	0.5780 (2)	−0.07062 (7)	0.0210 (3)	
C11	0.37468 (16)	0.7875 (3)	−0.07459 (8)	0.0334 (4)	
H11	0.3446	0.9122	−0.0861	0.040*	
C12	0.44090 (16)	0.7817 (3)	−0.00012 (8)	0.0327 (4)	
C13	0.46307 (17)	0.7337 (4)	−0.12182 (9)	0.0504 (6)	
H13A	0.5417	0.7993	−0.1089	0.076*	
H13B	0.4252	0.7625	−0.1693	0.076*	
H13C	0.4793	0.6047	−0.1177	0.076*	
C14	−0.16387 (14)	0.5988 (2)	−0.09751 (8)	0.0238 (3)	
H14A	−0.2067	0.6226	−0.0593	0.036*	
H14B	−0.1967	0.4884	−0.1209	0.036*	
H14C	−0.1778	0.6990	−0.1299	0.036*	
C15	0.28150 (14)	0.5536 (2)	0.15730 (7)	0.0232 (3)	
H15A	0.3241	0.4427	0.1469	0.028*	
H15B	0.3384	0.6554	0.1546	0.028*	
C16	0.14984 (13)	0.7902 (2)	−0.24440 (7)	0.0212 (3)	
C17	0.20561 (16)	0.7550 (2)	−0.30617 (7)	0.0298 (4)	
H17A	0.1449	0.7846	−0.3476	0.045*	
H17B	0.2283	0.6282	−0.3073	0.045*	
H17C	0.2803	0.8290	−0.3042	0.045*	
O1A	0.10059 (9)	0.30940 (15)	0.46612 (5)	0.0229 (2)	
O2A	−0.08856 (10)	0.34776 (18)	0.49033 (5)	0.0320 (3)	
O3A	0.61587 (9)	0.19216 (15)	0.43180 (5)	0.0258 (2)	
O4A	0.29523 (9)	0.22296 (15)	0.69401 (5)	0.0214 (2)	
O5A	0.45039 (11)	0.39546 (17)	0.74928 (5)	0.0320 (3)	
O6A	0.18713 (10)	0.21622 (16)	0.27100 (5)	0.0258 (2)	
H60A	0.2039 (19)	0.317 (3)	0.2552 (10)	0.039*	
C1A	0.44199 (13)	0.17797 (19)	0.49391 (7)	0.0188 (3)	
C2A	0.50439 (13)	0.1995 (2)	0.43344 (7)	0.0205 (3)	
C3A	0.40492 (14)	0.2229 (2)	0.37344 (7)	0.0215 (3)	
H3A	0.4187	0.2443	0.3283	0.026*	
C4A	0.29305 (13)	0.2106 (2)	0.39010 (7)	0.0199 (3)	
C5A	0.30176 (13)	0.1693 (2)	0.46618 (7)	0.0190 (3)	
H5A	0.2720	0.0442	0.4712	0.023*	
C6A	0.22888 (13)	0.2972 (2)	0.50361 (7)	0.0191 (3)	
H6A	0.2685	0.4184	0.5069	0.023*	
C7A	0.21326 (12)	0.2344 (2)	0.57504 (6)	0.0196 (3)	
H7A	0.1957	0.1028	0.5728	0.024*	
C8A	0.33015 (13)	0.2662 (2)	0.62853 (7)	0.0194 (3)	
H8A	0.3582	0.3935	0.6277	0.023*	
C9A	0.43475 (14)	0.1374 (2)	0.61948 (7)	0.0215 (3)	
H91A	0.4979	0.1383	0.6624	0.026*	
H92A	0.3995	0.0150	0.6138	0.026*	
C10A	0.50089 (13)	0.17411 (19)	0.56004 (7)	0.0193 (3)	
C11A	0.09343 (13)	0.3330 (2)	0.58339 (7)	0.0250 (3)	
H11A	0.1154	0.4596	0.5969	0.030*	
C12A	0.02146 (14)	0.3323 (2)	0.51022 (7)	0.0243 (3)	
C13A	0.01788 (14)	0.2562 (3)	0.63306 (8)	0.0307 (4)	
H13D	−0.0645	0.3130	0.6254	0.046*	
H13E	0.0609	0.2793	0.6802	0.046*	
H13F	0.0083	0.1268	0.6257	0.046*	
C14A	0.63888 (13)	0.2030 (2)	0.58228 (7)	0.0230 (3)	
H14D	0.6761	0.2265	0.5420	0.034*	
H14E	0.6768	0.0957	0.6059	0.034*	
H14F	0.6535	0.3057	0.6135	0.034*	
C15A	0.17124 (13)	0.2246 (2)	0.34040 (7)	0.0231 (3)	
H15C	0.1158	0.1259	0.3492	0.028*	
H15D	0.1303	0.3391	0.3481	0.028*	
C16A	0.36493 (14)	0.2941 (2)	0.75074 (7)	0.0233 (3)	
C17A	0.32219 (17)	0.2309 (3)	0.81365 (8)	0.0351 (4)	
H17D	0.3798	0.2738	0.8543	0.053*	
H17E	0.3202	0.0996	0.8140	0.053*	
H17F	0.2385	0.2776	0.8140	0.053*	
O7	0.29309 (12)	0.89082 (18)	0.25390 (6)	0.0333 (3)	
H72	0.371 (2)	0.911 (3)	0.2489 (11)	0.050 (6)*	
H71	0.259 (3)	0.997 (4)	0.2573 (14)	0.077 (9)*	

### Atomic displacement parameters (Å^2^)

**Table:**

	*U*^11^	*U*^22^	*U*^33^	*U*^12^	*U*^13^	*U*^23^
O1	0.0262 (5)	0.0319 (6)	0.0171 (5)	−0.0085 (5)	0.0001 (4)	0.0013 (4)
O2	0.0361 (7)	0.0768 (11)	0.0268 (6)	−0.0292 (7)	0.0006 (5)	0.0032 (7)
O3	0.0234 (5)	0.0289 (6)	0.0248 (5)	−0.0002 (4)	0.0058 (4)	−0.0015 (4)
O4	0.0331 (6)	0.0249 (6)	0.0143 (5)	0.0018 (5)	0.0053 (4)	0.0022 (4)
O5	0.0432 (7)	0.0384 (7)	0.0231 (5)	0.0132 (6)	0.0097 (5)	0.0106 (5)
O6	0.0346 (6)	0.0261 (6)	0.0145 (5)	0.0005 (5)	0.0035 (4)	0.0008 (4)
C1	0.0237 (8)	0.0170 (7)	0.0196 (7)	−0.0023 (6)	0.0025 (6)	0.0017 (6)
C2	0.0261 (8)	0.0147 (7)	0.0224 (7)	−0.0015 (6)	0.0041 (6)	0.0002 (6)
C3	0.0297 (8)	0.0192 (8)	0.0165 (7)	−0.0003 (6)	0.0055 (6)	−0.0001 (6)
C4	0.0268 (8)	0.0164 (7)	0.0154 (7)	0.0000 (6)	0.0032 (6)	0.0011 (6)
C5	0.0244 (7)	0.0184 (8)	0.0139 (7)	0.0004 (6)	0.0021 (5)	0.0003 (5)
C6	0.0224 (7)	0.0216 (8)	0.0142 (6)	−0.0019 (6)	−0.0020 (5)	0.0007 (6)
C7	0.0254 (7)	0.0225 (8)	0.0150 (6)	−0.0038 (6)	0.0035 (5)	0.0024 (6)
C8	0.0298 (7)	0.0210 (8)	0.0126 (6)	0.0001 (6)	0.0053 (5)	0.0000 (6)
C9	0.0247 (7)	0.0251 (8)	0.0149 (7)	−0.0012 (6)	−0.0003 (6)	−0.0013 (6)
C10	0.0261 (8)	0.0172 (7)	0.0191 (7)	−0.0012 (6)	0.0025 (6)	0.0003 (6)
C11	0.0371 (9)	0.0417 (10)	0.0199 (7)	−0.0173 (8)	0.0014 (6)	0.0049 (7)
C12	0.0346 (9)	0.0399 (10)	0.0225 (8)	−0.0161 (8)	0.0025 (7)	0.0027 (7)
C13	0.0353 (9)	0.0924 (17)	0.0248 (8)	−0.0282 (11)	0.0085 (7)	0.0021 (10)
C14	0.0266 (8)	0.0233 (8)	0.0198 (7)	−0.0003 (6)	0.0000 (6)	0.0008 (6)
C15	0.0267 (8)	0.0270 (8)	0.0154 (7)	0.0020 (6)	0.0029 (6)	0.0001 (6)
C16	0.0230 (7)	0.0226 (8)	0.0165 (7)	−0.0048 (6)	−0.0002 (5)	0.0024 (6)
C17	0.0395 (9)	0.0320 (10)	0.0186 (7)	0.0007 (7)	0.0070 (6)	0.0023 (6)
O1A	0.0214 (5)	0.0301 (6)	0.0166 (5)	0.0057 (4)	0.0020 (4)	0.0010 (4)
O2A	0.0241 (6)	0.0444 (7)	0.0266 (6)	0.0095 (5)	0.0026 (4)	0.0035 (5)
O3A	0.0242 (5)	0.0290 (6)	0.0256 (5)	0.0001 (5)	0.0086 (4)	0.0015 (5)
O4A	0.0243 (5)	0.0254 (5)	0.0145 (4)	−0.0015 (4)	0.0037 (4)	−0.0016 (4)
O5A	0.0313 (6)	0.0398 (7)	0.0234 (5)	−0.0079 (5)	0.0013 (4)	−0.0044 (5)
O6A	0.0361 (6)	0.0262 (6)	0.0147 (5)	0.0022 (5)	0.0042 (4)	−0.0005 (5)
C1A	0.0223 (7)	0.0138 (7)	0.0205 (7)	0.0005 (6)	0.0045 (5)	0.0003 (6)
C2A	0.0254 (7)	0.0155 (8)	0.0214 (7)	0.0001 (6)	0.0062 (5)	−0.0007 (6)
C3A	0.0301 (7)	0.0187 (7)	0.0165 (6)	−0.0012 (6)	0.0066 (5)	0.0000 (6)
C4A	0.0284 (7)	0.0144 (7)	0.0167 (6)	0.0002 (6)	0.0035 (5)	−0.0021 (6)
C5A	0.0227 (7)	0.0172 (7)	0.0169 (6)	0.0001 (6)	0.0029 (5)	0.0004 (6)
C6A	0.0193 (7)	0.0208 (7)	0.0162 (6)	0.0012 (6)	0.0008 (5)	0.0007 (6)
C7A	0.0214 (7)	0.0223 (8)	0.0152 (6)	−0.0007 (6)	0.0038 (5)	−0.0006 (6)
C8A	0.0226 (7)	0.0225 (8)	0.0132 (6)	−0.0001 (6)	0.0037 (5)	0.0007 (5)
C9A	0.0236 (7)	0.0225 (8)	0.0178 (7)	0.0016 (6)	0.0019 (5)	0.0014 (6)
C10A	0.0235 (7)	0.0140 (7)	0.0208 (7)	0.0027 (6)	0.0052 (5)	−0.0004 (6)
C11A	0.0233 (7)	0.0327 (9)	0.0184 (7)	0.0047 (7)	0.0021 (6)	−0.0023 (6)
C12A	0.0266 (8)	0.0247 (8)	0.0220 (7)	0.0054 (6)	0.0056 (6)	−0.0002 (6)
C13A	0.0263 (8)	0.0437 (11)	0.0233 (7)	0.0063 (7)	0.0080 (6)	0.0039 (7)
C14A	0.0247 (7)	0.0212 (8)	0.0224 (7)	−0.0011 (6)	0.0029 (5)	−0.0007 (6)
C15A	0.0283 (8)	0.0249 (8)	0.0157 (6)	0.0001 (7)	0.0035 (5)	−0.0009 (6)
C16A	0.0250 (7)	0.0257 (8)	0.0183 (7)	0.0049 (7)	0.0013 (6)	−0.0024 (6)
C17A	0.0450 (9)	0.0429 (10)	0.0175 (7)	−0.0045 (9)	0.0059 (6)	−0.0025 (7)
O7	0.0351 (7)	0.0283 (7)	0.0353 (6)	0.0043 (6)	0.0031 (5)	−0.0038 (5)

### Geometric parameters (Å, °)

**Table:**

O1—C12	1.3568 (19)	O1A—C6A	1.4612 (16)
O1—C6	1.4597 (16)	O2A—C12A	1.1982 (19)
O2—C12	1.194 (2)	O3A—C2A	1.2259 (18)
O3—C2	1.2251 (19)	O4A—C16A	1.3445 (18)
O4—C16	1.3496 (18)	O4A—C8A	1.4586 (16)
O4—C8	1.4598 (16)	O5A—C16A	1.207 (2)
O5—C16	1.196 (2)	O6A—C15A	1.4224 (16)
O6—C15	1.4238 (17)	O6A—H60A	0.85 (2)
O6—H6O	0.86 (2)	C1A—C10A	1.349 (2)
C1—C10	1.349 (2)	C1A—C2A	1.4969 (19)
C1—C2	1.500 (2)	C1A—C5A	1.5291 (19)
C1—C5	1.5264 (19)	C2A—C3A	1.4644 (19)
C2—C3	1.463 (2)	C3A—C4A	1.328 (2)
C3—C4	1.334 (2)	C3A—H3A	0.9500
C3—H3	0.9500	C4A—C15A	1.5041 (19)
C4—C15	1.500 (2)	C4A—C5A	1.5271 (18)
C4—C5	1.5261 (19)	C5A—C6A	1.5238 (19)
C5—C6	1.525 (2)	C5A—H5A	1.0000
C5—H5	1.0000	C6A—C7A	1.5337 (18)
C6—C7	1.5323 (18)	C6A—H6A	1.0000
C6—H6	1.0000	C7A—C8A	1.5182 (19)
C7—C8	1.5177 (19)	C7A—C11A	1.538 (2)
C7—C11	1.527 (2)	C7A—H7A	1.0000
C7—H7	1.0000	C8A—C9A	1.530 (2)
C8—C9	1.531 (2)	C8A—H8A	1.0000
C8—H8	1.0000	C9A—C10A	1.5206 (19)
C9—C10	1.516 (2)	C9A—H91A	0.9900
C9—H9A	0.9900	C9A—H92A	0.9900
C9—H9B	0.9900	C10A—C14A	1.505 (2)
C10—C14	1.502 (2)	C11A—C13A	1.513 (2)
C11—C12	1.520 (2)	C11A—C12A	1.517 (2)
C11—C13	1.521 (3)	C11A—H11A	1.0000
C11—H11	1.0000	C13A—H13D	0.9800
C13—H13A	0.9800	C13A—H13E	0.9800
C13—H13B	0.9800	C13A—H13F	0.9800
C13—H13C	0.9800	C14A—H14D	0.9800
C14—H14A	0.9800	C14A—H14E	0.9800
C14—H14B	0.9800	C14A—H14F	0.9800
C14—H14C	0.9800	C15A—H15C	0.9900
C15—H15A	0.9900	C15A—H15D	0.9900
C15—H15B	0.9900	C16A—C17A	1.490 (2)
C16—C17	1.492 (2)	C17A—H17D	0.9800
C17—H17A	0.9800	C17A—H17E	0.9800
C17—H17B	0.9800	C17A—H17F	0.9800
C17—H17C	0.9800	O7—H72	0.89 (2)
O1A—C12A	1.3524 (18)	O7—H71	0.89 (3)
			
C12—O1—C6	109.37 (11)	C16A—O4A—C8A	117.23 (11)
C16—O4—C8	117.62 (11)	C15A—O6A—H60A	113.1 (14)
C15—O6—H6O	107.0 (14)	C10A—C1A—C2A	125.23 (13)
C10—C1—C2	126.38 (13)	C10A—C1A—C5A	127.71 (13)
C10—C1—C5	126.46 (13)	C2A—C1A—C5A	107.01 (11)
C2—C1—C5	107.11 (12)	O3A—C2A—C3A	124.94 (13)
O3—C2—C3	124.87 (13)	O3A—C2A—C1A	128.43 (13)
O3—C2—C1	128.30 (13)	C3A—C2A—C1A	106.56 (11)
C3—C2—C1	106.82 (12)	C4A—C3A—C2A	111.47 (12)
C4—C3—C2	111.18 (13)	C4A—C3A—H3A	124.3
C4—C3—H3	124.4	C2A—C3A—H3A	124.3
C2—C3—H3	124.4	C3A—C4A—C15A	125.03 (12)
C3—C4—C15	125.26 (13)	C3A—C4A—C5A	111.81 (12)
C3—C4—C5	111.98 (13)	C15A—C4A—C5A	123.08 (12)
C15—C4—C5	122.64 (13)	C6A—C5A—C4A	114.51 (12)
C6—C5—C4	114.20 (12)	C6A—C5A—C1A	112.21 (11)
C6—C5—C1	109.34 (11)	C4A—C5A—C1A	102.67 (11)
C4—C5—C1	102.88 (11)	C6A—C5A—H5A	109.1
C6—C5—H5	110.1	C4A—C5A—H5A	109.1
C4—C5—H5	110.1	C1A—C5A—H5A	109.1
C1—C5—H5	110.1	O1A—C6A—C5A	109.26 (11)
O1—C6—C5	111.28 (11)	O1A—C6A—C7A	103.13 (11)
O1—C6—C7	103.45 (11)	C5A—C6A—C7A	114.76 (12)
C5—C6—C7	114.66 (12)	O1A—C6A—H6A	109.8
O1—C6—H6	109.1	C5A—C6A—H6A	109.8
C5—C6—H6	109.1	C7A—C6A—H6A	109.8
C7—C6—H6	109.1	C8A—C7A—C6A	111.94 (11)
C8—C7—C11	117.26 (12)	C8A—C7A—C11A	118.10 (12)
C8—C7—C6	112.75 (12)	C6A—C7A—C11A	101.45 (11)
C11—C7—C6	101.19 (12)	C8A—C7A—H7A	108.3
C8—C7—H7	108.4	C6A—C7A—H7A	108.3
C11—C7—H7	108.4	C11A—C7A—H7A	108.3
C6—C7—H7	108.4	O4A—C8A—C7A	105.20 (11)
O4—C8—C7	106.51 (11)	O4A—C8A—C9A	107.00 (11)
O4—C8—C9	107.23 (11)	C7A—C8A—C9A	111.62 (12)
C7—C8—C9	111.92 (12)	O4A—C8A—H8A	110.9
O4—C8—H8	110.4	C7A—C8A—H8A	110.9
C7—C8—H8	110.4	C9A—C8A—H8A	110.9
C9—C8—H8	110.4	C10A—C9A—C8A	116.93 (12)
C10—C9—C8	114.89 (12)	C10A—C9A—H91A	108.1
C10—C9—H9A	108.5	C8A—C9A—H91A	108.1
C8—C9—H9A	108.5	C10A—C9A—H92A	108.1
C10—C9—H9B	108.5	C8A—C9A—H92A	108.1
C8—C9—H9B	108.5	H91A—C9A—H92A	107.3
H9A—C9—H9B	107.5	C1A—C10A—C14A	123.41 (13)
C1—C10—C14	123.54 (14)	C1A—C10A—C9A	123.36 (13)
C1—C10—C9	122.87 (13)	C14A—C10A—C9A	113.23 (11)
C14—C10—C9	113.58 (12)	C13A—C11A—C12A	112.04 (13)
C12—C11—C13	110.55 (15)	C13A—C11A—C7A	117.99 (14)
C12—C11—C7	101.57 (12)	C12A—C11A—C7A	101.17 (11)
C13—C11—C7	117.49 (16)	C13A—C11A—H11A	108.4
C12—C11—H11	108.9	C12A—C11A—H11A	108.4
C13—C11—H11	108.9	C7A—C11A—H11A	108.4
C7—C11—H11	108.9	O2A—C12A—O1A	121.52 (13)
O2—C12—O1	121.59 (14)	O2A—C12A—C11A	128.52 (14)
O2—C12—C11	128.38 (15)	O1A—C12A—C11A	109.96 (12)
O1—C12—C11	110.02 (13)	C11A—C13A—H13D	109.5
C11—C13—H13A	109.5	C11A—C13A—H13E	109.5
C11—C13—H13B	109.5	H13D—C13A—H13E	109.5
H13A—C13—H13B	109.5	C11A—C13A—H13F	109.5
C11—C13—H13C	109.5	H13D—C13A—H13F	109.5
H13A—C13—H13C	109.5	H13E—C13A—H13F	109.5
H13B—C13—H13C	109.5	C10A—C14A—H14D	109.5
C10—C14—H14A	109.5	C10A—C14A—H14E	109.5
C10—C14—H14B	109.5	H14D—C14A—H14E	109.5
H14A—C14—H14B	109.5	C10A—C14A—H14F	109.5
C10—C14—H14C	109.5	H14D—C14A—H14F	109.5
H14A—C14—H14C	109.5	H14E—C14A—H14F	109.5
H14B—C14—H14C	109.5	O6A—C15A—C4A	112.23 (12)
O6—C15—C4	112.58 (12)	O6A—C15A—H15C	109.2
O6—C15—H15A	109.1	C4A—C15A—H15C	109.2
C4—C15—H15A	109.1	O6A—C15A—H15D	109.2
O6—C15—H15B	109.1	C4A—C15A—H15D	109.2
C4—C15—H15B	109.1	H15C—C15A—H15D	107.9
H15A—C15—H15B	107.8	O5A—C16A—O4A	123.13 (14)
O5—C16—O4	123.69 (13)	O5A—C16A—C17A	125.78 (14)
O5—C16—C17	125.11 (14)	O4A—C16A—C17A	111.09 (13)
O4—C16—C17	111.18 (13)	C16A—C17A—H17D	109.5
C16—C17—H17A	109.5	C16A—C17A—H17E	109.5
C16—C17—H17B	109.5	H17D—C17A—H17E	109.5
H17A—C17—H17B	109.5	C16A—C17A—H17F	109.5
C16—C17—H17C	109.5	H17D—C17A—H17F	109.5
H17A—C17—H17C	109.5	H17E—C17A—H17F	109.5
H17B—C17—H17C	109.5	H72—O7—H71	106 (2)
C12A—O1A—C6A	110.30 (10)		
			
C10—C1—C2—O3	1.9 (3)	C10A—C1A—C2A—O3A	11.1 (2)
C5—C1—C2—O3	179.40 (15)	C5A—C1A—C2A—O3A	−171.14 (15)
C10—C1—C2—C3	−179.34 (14)	C10A—C1A—C2A—C3A	−171.77 (15)
C5—C1—C2—C3	−1.83 (16)	C5A—C1A—C2A—C3A	6.04 (16)
O3—C2—C3—C4	−179.83 (14)	O3A—C2A—C3A—C4A	174.67 (15)
C1—C2—C3—C4	1.35 (17)	C1A—C2A—C3A—C4A	−2.63 (18)
C2—C3—C4—C15	−176.29 (14)	C2A—C3A—C4A—C15A	−178.68 (14)
C2—C3—C4—C5	−0.30 (18)	C2A—C3A—C4A—C5A	−1.99 (18)
C3—C4—C5—C6	117.53 (15)	C3A—C4A—C5A—C6A	127.48 (14)
C15—C4—C5—C6	−66.36 (18)	C15A—C4A—C5A—C6A	−55.76 (19)
C3—C4—C5—C1	−0.85 (16)	C3A—C4A—C5A—C1A	5.58 (16)
C15—C4—C5—C1	175.27 (13)	C15A—C4A—C5A—C1A	−177.65 (13)
C10—C1—C5—C6	57.39 (19)	C10A—C1A—C5A—C6A	47.5 (2)
C2—C1—C5—C6	−120.12 (13)	C2A—C1A—C5A—C6A	−130.28 (12)
C10—C1—C5—C4	179.11 (15)	C10A—C1A—C5A—C4A	170.90 (15)
C2—C1—C5—C4	1.60 (15)	C2A—C1A—C5A—C4A	−6.84 (15)
C12—O1—C6—C5	149.85 (14)	C12A—O1A—C6A—C5A	146.40 (12)
C12—O1—C6—C7	26.24 (17)	C12A—O1A—C6A—C7A	23.91 (15)
C4—C5—C6—O1	48.63 (17)	C4A—C5A—C6A—O1A	52.01 (16)
C1—C5—C6—O1	163.25 (11)	C1A—C5A—C6A—O1A	168.54 (11)
C4—C5—C6—C7	165.59 (12)	C4A—C5A—C6A—C7A	167.24 (12)
C1—C5—C6—C7	−79.78 (15)	C1A—C5A—C6A—C7A	−76.23 (15)
O1—C6—C7—C8	−163.07 (12)	O1A—C6A—C7A—C8A	−162.59 (12)
C5—C6—C7—C8	75.57 (16)	C5A—C6A—C7A—C8A	78.68 (16)
O1—C6—C7—C11	−36.96 (15)	O1A—C6A—C7A—C11A	−35.75 (14)
C5—C6—C7—C11	−158.32 (13)	C5A—C6A—C7A—C11A	−154.48 (12)
C16—O4—C8—C7	−135.67 (13)	C16A—O4A—C8A—C7A	−158.28 (12)
C16—O4—C8—C9	104.36 (14)	C16A—O4A—C8A—C9A	82.89 (15)
C11—C7—C8—O4	56.71 (17)	C6A—C7A—C8A—O4A	172.54 (11)
C6—C7—C8—O4	173.63 (12)	C11A—C7A—C8A—O4A	55.30 (16)
C11—C7—C8—C9	173.60 (13)	C6A—C7A—C8A—C9A	−71.77 (16)
C6—C7—C8—C9	−69.48 (16)	C11A—C7A—C8A—C9A	170.99 (13)
O4—C8—C9—C10	−166.64 (12)	O4A—C8A—C9A—C10A	−170.91 (12)
C7—C8—C9—C10	76.91 (16)	C7A—C8A—C9A—C10A	74.50 (16)
C2—C1—C10—C14	−2.7 (2)	C2A—C1A—C10A—C14A	4.0 (2)
C5—C1—C10—C14	−179.77 (14)	C5A—C1A—C10A—C14A	−173.31 (14)
C2—C1—C10—C9	178.21 (14)	C2A—C1A—C10A—C9A	−175.04 (13)
C5—C1—C10—C9	1.2 (2)	C5A—C1A—C10A—C9A	7.6 (2)
C8—C9—C10—C1	−60.8 (2)	C8A—C9A—C10A—C1A	−59.5 (2)
C8—C9—C10—C14	120.02 (14)	C8A—C9A—C10A—C14A	121.36 (14)
C8—C7—C11—C12	156.71 (14)	C8A—C7A—C11A—C13A	−80.60 (18)
C6—C7—C11—C12	33.66 (17)	C6A—C7A—C11A—C13A	156.70 (14)
C8—C7—C11—C13	−82.6 (2)	C8A—C7A—C11A—C12A	156.85 (13)
C6—C7—C11—C13	154.36 (15)	C6A—C7A—C11A—C12A	34.14 (15)
C6—O1—C12—O2	175.77 (18)	C6A—O1A—C12A—O2A	179.11 (15)
C6—O1—C12—C11	−4.1 (2)	C6A—O1A—C12A—C11A	−1.34 (17)
C13—C11—C12—O2	35.0 (3)	C13A—C11A—C12A—O2A	31.4 (2)
C7—C11—C12—O2	160.5 (2)	C7A—C11A—C12A—O2A	157.96 (17)
C13—C11—C12—O1	−145.13 (17)	C13A—C11A—C12A—O1A	−148.13 (14)
C7—C11—C12—O1	−19.7 (2)	C7A—C11A—C12A—O1A	−21.55 (16)
C3—C4—C15—O6	6.5 (2)	C3A—C4A—C15A—O6A	12.0 (2)
C5—C4—C15—O6	−169.07 (13)	C5A—C4A—C15A—O6A	−164.31 (13)
C8—O4—C16—O5	−1.4 (2)	C8A—O4A—C16A—O5A	2.9 (2)
C8—O4—C16—C17	177.21 (12)	C8A—O4A—C16A—C17A	−177.12 (12)

### Hydrogen-bond geometry (Å, °)

**Table:**

*D*—H···*A*	*D*—H	H···*A*	*D*···*A*	*D*—H···*A*
O6—H6O···O7	0.86 (2)	1.85 (2)	2.6750 (17)	160 (2)
O6A—H60A···O6	0.85 (2)	1.91 (2)	2.7593 (17)	174 (2)
O7—H72···O5A^i^	0.89 (2)	1.95 (2)	2.8160 (18)	165 (2)
O7—H71···O6A^ii^	0.89 (3)	1.86 (3)	2.7397 (18)	176 (3)

Symmetry codes: (i) −*x*+1, *y*+1/2, −*z*+1; (ii) *x*, *y*+1, *z*.
